# Treatment of depression with Chai Hu Shu Gan San: a systematic review and meta-analysis of 42 randomized controlled trials

**DOI:** 10.1186/s12906-018-2130-z

**Published:** 2018-02-17

**Authors:** Yan Sun, Xia Xu, Jinping Zhang, Yuanyuan Chen

**Affiliations:** 1 Graduated School, Tianjin University of Chinese Medicine, Tianjin, 300193 China; 2 Department of Chinese Medicine, Nankai Hospital, No. 6 Changjiang Road, Nankai District, Tianjin, 300100 China; 3 Graduated School, Tianjin University of Chinese Medicine, Tianjin, 300193 China; 4 Department of Chinese Medicine, Nankai Hospital, Tianjin, 300100 China; 5 Department of Pharmacy, Affiliated Hospital of Logistics Institute of Chinese Armed Police Force, Tianjin, 300163 China

**Keywords:** Chai Hu Shu Gan san, Depression, Randomized control trial, Systematic review

## Abstract

**Background:**

Depression is a common mental disorder. Chai Hu Shu Gan San, a traditional Chinese medicine, is used to treat depression empirically. We present a systematic review and meta-analysis of the therapeutic efficacy and safety of Chai Hu Shu Gan San in treating depression.

**Methods:**

Several databases, including PubMed, China National Knowledge Internet, Wanfang, Chongqing VIP, and the Cochrane library, were systematically searched from their date of foundation to January 1, 2017. In this review, wehave included randomized control trials that compared Chai Hu Shu Gan San (or its combination with a regular Western medicine) with a regular Western medicine alone for the treatment of depression. Two investigators independently extracted and analyzed the data using RevMan 5.2.0 software. Mean difference (with a 95% confidence interval) was used as efficacy indices for outcomes.

**Results:**

We included 42 studies involving 3234 patients with depression in 15 different types of diseases. Meta analyses showed better effect of Chai Hu Shu Gan San than fluoxetine for pure depression (MD = − 1.59, from − 2.82 to − 0.37, 4 trials, I^2^ = 26%), for post-stroke depression (MD = − 4.20, from − 6.20 to − 2.19, 7 trials, I^2^ = 96%), and for postpartum depression (MD = − 4.10, from − 7.48 to − 0.72 7 trials, I^2^ = 86%). None of the articles reported severe adverse events of oral administration of Chai Hu Shu Gan San. Furthermore, any adverse effects of using Chai Hu Shu Gan San alone were fewer than those of regular Western medicines.

**Conclusions:**

This review found that Chai Hu Shu Gan San has some advantages in treating depression, especially post-stroke depression and post-partum depression. A meticulously designed and conducted randomized control trial is needed for further evaluation.

## Background

Depression is a common mental disorder primarily characterized by the presence of low spirit, anhedonia, insomnia, loss of appetite, inattention, and even suicide [1]. In high-income countries, the prevalence depression is approximately 5.1% [2], and the annual incidence of depression is approximately 3% [3]. Some studies predict that depression will be the main factor leading to death and disability in high-income countries by the year 2030 [4]. To date, anti-depressant drugs commonly used in clinics are primarily categorized into two types: a selective serotonin-norepinephrine reuptake inhibitor and a selective 5-hydroxy tryptamine reuptake inhibitor. Although these two types of inhibitors have definite therapeutic efficacies, their long-term usage has severe adverse effects, leading to poor compliance [5] and subsequent relapse of depression. Therefore, China and other countries are increasing their efforts toidentify traditional Chinese medicines with less adverse effects.

With a deeper understanding of depression in traditional Chinese medicine and in the light of traditional Chinese medicine theories, Chai Hu Shu Gan San is being increasingly used to treat depression. Chai Hu Shu Gan San is described in the Chinese ancient book *Jing Yue Quan Shu • Gu Fang Ba Zhen • San Zhen*, by Jingyue Zhang of the Ming dynasty. The primary constituent of this formula is Chai Hu (*Bupleurum Chinese*). The formula has two other constituents: Xiangfu (*Cyperus rotundus*) and Chuanxiong (*Ligusticum chuanxiong*). In China, a large number of clinical trial studies have indicated that Chai Hu Shu Gan San is widely used for treating all types of depression, however, no publication has been identified to summarize the evidence and evaluate its effect and safety. This study, therefore, aims to systematically review the efficacy and safety of Chai Hu Shu Gan San for the treatment of depression, thereby laying an evidence-based foundation for clinical therapy.

## Methods

### Criteria for inclusion and exclusion

The criteria for inclusion were as follows: (1) study types: a randomized control trial (RCT)—regardless of blind methods and placebos—that was reported in either Chinese or English; (2) participants: patients with definite diagnosis of depression (Major depressive disorder, MDD), irrespective of the cause or presence of other diseases; (3) interventions: test groups were treated with Chai Hu Shu Gan Yin (San) alone, without restriction of its doses and therapeutic periods, and control groups were treated with placebos, regular Western medicines, or no therapy at all;participants with any concomitant disease in both a test group and a control group were simultaneously subjected to the same treatments, other than the above-mentioned ones, regardless of therapeutic periods, therapeutic methods, and medicine doses; (4) outcome index: the Hamilton Depression Scale (HAMD) scores of the patients, the efficacy rate of the medicines, and theadverseeventsof medicines.

We excluded articles that RCT that did not report outcome indices relevant to this study.

### Search strategy

Reference searches in PubMed, the Cochrane library, Chongqing VIP database, China National Knowledge Internet (CNKI), and Wanfang database were conducted. The following search terms were included: “Chai Hu Shu Gan”, “depression”, and “random”.The above terms in Chinese were adapted and searched in Chinese databases. The search period was from the time of establishment of each database to January 1, 2017, and the references cited by the retrieved articles were tracked.

### Reference screening

For all of the retrieved references, two independent researchers (Yan Sun and Xia Xu) read the titles and abstracts and excluded any studies in which the RCT failed to fulfill the criteria for inclusion. The researchers further read the full text of the remaining articles to determine whether they fulfilled the criteria for inclusion. They then cross-verified their conclusions. If it was difficult for the two researchers to reach a consensus on whether a study should be included, the disagreement was resolved after discussion with a third party.

### Methodological quality assessment for the included studies

The methodological quality of the RCTs wasassessed using methods recommended by the Cochrane Collaboration. This approachprimarily involves a risk/bias assessment based onsix items [6]:the generation of random sequences, concealment of random allocation, blinding method, data completeness, selective reporting of outcomes, and estimation of sample size. If a study fulfilled each aforementioned item, it had a low risk of bias; otherwise, it had a high risk of bias. When it was impossible to assess whether an article fulfilled the aforementioned criteria, because of insufficient information in the article, the study was considered “unclear”. The methodological quality assessment of clinical trials was conducted by the two independent researchers (Jinping Zhang and Yuanyuan Chen), and any disagreements were resolved after a discussion with a third party.

### Information extraction and analysis

The two researchers used the same information-extraction table to independently extract the information, which primarily included titles, general characteristics of the patients, concomitant diseases, intervention and control measures, follow-up, and indices for the assessment of therapeutic efficacy.

A meta-analysis was conducted with RevMan 5.2.0 software, which was provided by the Cochrane Collaboration. The count data was expressed in terms of odd ratio (OR), and the measurement data was expressed in terms of mean difference (MD) with a 95% confidence interval (CI). The effects were expressed with the random-effects model (REM).

## Results

### Procedure for study inclusion

**Fig. 1:**
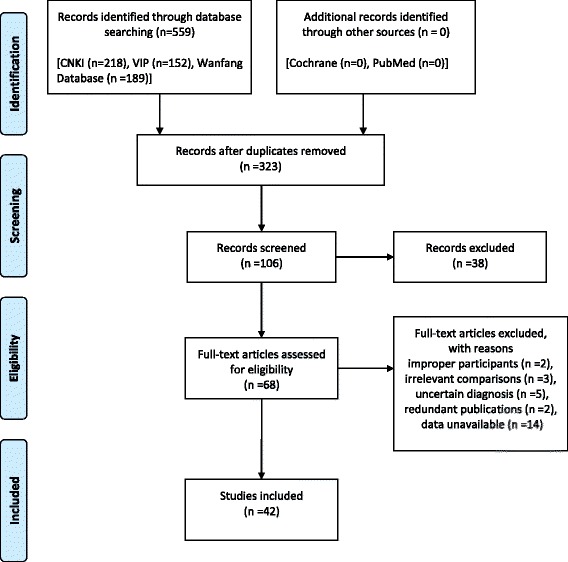
PRISMA 2009 Flow Diagram

In this review, we initially identified 560 relevant studies from 5 databases and 323 duplicate records were removed. The remaining 106 records were screened, in which 38 records were excluded by reading titles and abstracts, 68 records were assessed in full texts, an additional 26 records were excluded for improper participants, irrelevant comparisons, single author, uncertain diagnosis, poor data authenticity, redundant publications, no RCTs or missing data unavailable from contacting the author. 42 trials met the inclusion criteria and were included in the meta-analysis [7–48]. The screening procedure is illustrated in Fig. 1.

### Description of studies

The 42 studies included in this review were all conducted and published in Chinese from 2006 to 2016. Together, these studies included 3234 patients with depression (consisting of 1784 patients in test groups and 1450 patients in control groups). Among the included investigations, two reported using three groups [16, 21], while the others reported using two-group parallel control methods. Thirteen studies investigated patients with pure depression [9, 18, 19, 23, 32, 34, 35, 37, 40, 43, 44, 46, 47], two studies investigated patients with post-partum depression (PD) [27, 28], while 27 studies investigated patients with other diseases concomitant with depression (consisting of eleven studies about post-stroke depression (PSD) [7, 8, 10, 12, 13, 15, 17, 22, 39, 42, 48], three about Parkinson’s disease concomitant with depression [20, 30, 41], one each about cancer concomitant with depression [11], seizures concomitant with depression [14], chronic pelvic inflammation concomitant with depression [16], post-percutaneous coronary intervention depression [24], two studies on digestive diseases concomitant with depression [21, 29], and two study on cerebral vascular disease concomitant with depression [26, 33], one study each about chronic obstructive pulmonary disease concomitant with depression [25], diabetes concomitant with depression [38], coronary heart disease concomitant with depression [31], rheumatoid arthritis concomitant with depression [36] and Cardiac neurosis concomitant with depression [45]).

Among the included studies in this review, 26 reported therapy for depression with Chai Hu Shu Gan San based on the traditional Chinese medicine hypothesis about syndrome differentiation and treatment, and others reported personalized therapies for depression without using this hypothesis. In 21 studies, treatment of depression was conducted with Chai Hu Shu Gan San alone. In the other studies, treatments involving a combination of Chai Hu Shu Gan San and Western medicines (fluoxetine in seven studies [7, 9, 12, 15, 17, 27, 28, 47], escitalopram oxalate in one study [10, 40], venlafaxine hydrochloride in one study [19], paroxetine hydrochloride in two study [22, 41], mirtazapine in two study [26, 34], duloxetine in one study [30], deanxit in three study [13, 42, 45], and sertraline hydrochloride in one study [20]) were investigated. None of the included studies used placebo controls. The therapeutic efficacy in a test group treated with Chai Hu Shu Gan San was compared with that of a control group (no treatment with any therapy in four studies [16, 21, 25, 29, 31], treated with fluoxetine in 16 studies [7–9, 11, 12, 15, 17, 18, 27, 28, 32, 33, 39, 43, 47, 48], paroxetine in ten studies [14, 16, 22, 23, 35, 36, 38, 41, 44, 46], deanxit therapy in five studies [13, 21, 24, 42, 45], and escitalopram oxalate in two studies [10, 40], mirtazapine in two studies [26, 34], sertraline hydrochloride [20], duloxetine [30], amitriptyline [37] and venlafaxine therapy [19] in one study each). Thirty-eight of the included studies employed the HAMD scale, with 33 reporting therapeutic efficacy for depression and 22 reporting the safety of the medications. No study reported the long-term effects by conducting follow-up surveys with subjects.

The characteristics of the included articles are shown in Table 1.

**Table 1:** Characteristics of the enrolled randomized controlled trials

Study ID	Sample size (I/C)	Gender (M/F)	Age	Intervention Group	Controlled Group	Syndrome Differentiation	Course of Treatment	Following-up	Outcomes		
									HAMD Mean score (SD)	Effective Rate (events/total)	Adverse Effect
Depression											
Cheng XY 2007 [7]	33/30	I:18/15; C:16/14	I:37.1 ± 7.6; C:36.7 ± 8.7	Chai Hu Shu Gan San + Fluoxetine	Fluoxetine	Y	6 weeks	N	6.32(2.33) vs 7.01(3.45)	NR	I: somnolence, dry mouth and sleepy; C: insomnia, blurred vision, nausea, headache. (Case unknown)
Lin B 2011 [8]	30/30	I:13/17; C:15/15	I:52.13 ± 4.31; C:50.43 ± 4.80	Chai Hu Shu Gan San	Fluoxetine	Y	20 days	N	11.53(7.41) vs 13.23(6.99)	26/30 vs 5/30	NR
Liu YY 2012 [9]	31/32	I:11/19; C:12/20	I:40.5 ± 9.9; C:36.6 ± 15.0	Chai Hu Shu Gan San + Venlafaxine	Venlafaxine	Y	4 weeks	N	5.9(4.6) vs 8(5.6)	NR	NR
Wang RC 2013 [10]	40/40	I:18/22; C:21/19	I:33.6 ± 10.75; C:34.70 ± 11.23	Chai Hu Shu Gan San	Paroxetine	Y	6 weeks	N	7.21(4.23) vs 7.52(3.79)	33/40 vs 34/40	I: slight headache, tiredness,constipation, sweat, bitter taste; C: dry mouth, constipation, excitement and agitation, insomnia, dizziness, headache, palpitation, quiver, nausea, vomit, sexuality descent, ejaculation inhibition, female sexual lack. (Case unknown)
Shao XQ 2016 [11]	15/13	I:8/7; C:7/6	I:37.4 ± 7.53; C:36.7 ± 7.61	Chai Hu Shu Gan San	Fluoxetine	Y	6 weeks	6 months	9.51(4.84) vs 10.24(4.01)	14/15 vs 12/13	No adverse effect reported in intervention group;Nausea, anorexia, headache, sexual dysfunction reported in control group (Case unknown)
Gu XX 2016 [12]	30/30	I:18/12 C:16/14	I:33.1 ± 14.4 C:32.8 ± 17.1	Chai Hu Shu Gan San + Mirtazapine	Mirtazapine	N	8 weeks	N	7.82(1.56) vs 15.88 (1.42)	27/30 vs 24/30	Appetite increased, weight gain, edema, nausea, dry mouth, sleep disorders (Case unknown)
Hu J 2015 [13]	48/48	I:20/28 C:22/26	I:38.56 ± 12.23 C:39.89 ± 11.83	Chai Hu Shu Gan San	Paroxetine	N	6 weeks	N	9.65(3.44) vs C:8.98(4.32)	43/48 vs 42/48	NR
Liu CY 2015 [14]	35/34	I:21/14C:19/15	I:49.12 ± 7.64 C:48.46 ± 7.25	Chai Hu Shu Gan San + Amitriptyline	Amitriptyline	Y	3 months	N	4.83(1.37) vs 4.79(1.02)	33/35 vs 28/34	constipation (I = 1 vs C = 7/34); dry mouth (I = 0 vs C = 4); dizzy(I = 1 vs C = 5);electrocardiographic abnormality (I = 1 vs C = 6)
Deng SZ 2012 [15]	53/48	I:28/25; C:23/25	NA	Chai Hu Shu Gan San + Citalopram	Citalopram	Y	8 weeks	N	6.07(1.86) vs 9.38(2.27)	49/53 vs 37/48	NR
Song YM 2011 [16]	30/30	I:11/19; C:12/18	I:46.5 ± 6.3; C:45.3 ± 7.2	Chai Hu Shu Gan San	Fluoxetine	Y	4 weeks	N	14.8(3.3) vs 17.6(2.8)	28/30 vs 24/30	NR
Fan QL 2008 [17]	70/35	I:28/42; C:13/22	I:67.5; C:67.5	Chai Hu Shu Gan San	Paroxetine	Y	3 months	N	NR	69/70 vs 29/35	NR
Deng GQ 2013 [18]	30/30	I:13/17; C:12/18	I:38.5 ± 10.4; C:40.2 ± 12.1	Chai Hu Shu Gan San	Paroxetine	Y	6 weeks	N	8.9(3.5) vs 9.7(2.8)	24/30 vs 22/30	NR
Wang L 2012 [19]	30/30	I:12/18; C:14/16	NA	Chai Hu Shu Gan San + Fluoxetine	Fluoxetine	Y	4 weeks	N	NR	28/30 vs 25/30	Insomnia, mental tension, nausea, headache (C = 9 vs I = 3)
Post-stroke Depression											
Chang XH 2010 [20]	50/50	I:31/19; C:28/22	I:42–74; C:45–75	Chai Hu Shu Gan San + Fluoxetine	Fluoxetine	Y	28 days	N	10.24(3.4) vs 14.2(2.7)	48/50 vs 41/50	NR
Chen HH 2013 [21]	47/47	I:30/17; C:29/18	I:67 ± 4; C:66 ± 5	Chai Hu Shu Gan San	Fluoxetine	N	4 weeks	N	5.82(1.56) vs 6.21(1.38)	43/47 vs 42/47	Nausea (I = 1 vs C = 14), nodal tachycardia (I = 0 vs C = 5), stomach discomfort (I = 2 vs C = 15), dry mouth (I = 2 vs C = 18), somnolence (I = 1 vs C = 15);
Cui Y 2016 [22]	30/30	I:18/12; C:16/14	I:52.23 ± 9.90; C:50.73 ± 10.52	Chai Hu Shu Gan San + Escitalopram Oxalate Tablets	Escitalopram Oxalate Tablets	Y	6 weeks	N	8.67(4.97) vs 12.4(6.97)	NR	no apparent adverse effect in both group.
He XM 2007 [23]	36/18	I:21/15; C:11/7	I:53.24 ± 6.31; C:54.36 ± 4.42	Chai Hu Shu Gan San + Fluoxetine	Fluoxetine	N	60 days	N	16.41(2.56) vs 22.06(3.35)	32/36 vs 11/18	digestive discomfort (I = 8 vs C = 10), vegetative nerve functional disturbance (I = 10 vs C = 9);
Huang WX 2010 [24]	32/31	I:17/15; C:16/15	I:65 ± 4.6; C:61 ± 5.3	Chai Hu Shu Gan San + Deanxit	Deanxit	Y	8 weeks	N	15.6(4.4) vs 16.2(4.9)	NR	I; dizziness (2 cases), constipation (1 cases); C: No adverse effect
Huang YS 2012 [25]	39/39	I:22/17; C:20/19	I:62.51 ± 7.47;C:61.93 ± 7.82	Chai Hu Shu Gan San + Fluoxetine	Fluoxetine	Y	3 months	N	9.57(2.11) vs 13.08(2.58)	35/39 vs 32/39	I: No adverse effect; C: 6 cases with dry mouth, nausea, vomit, appetite reduced, insomnia, headache, tiredness.
Lian Z 2009 [26]	30/30	I:17/13; C:16/14	I:56.20 ± 18.6; C:54.6 ± 17.5	Chai Hu Shu Gan San + Fluoxetine	Fluoxetine	N	60 days	N	6.21(2.53) vs 12.1(1.25)	26/30 vs 24/30	gastrointestinal discomfort (I = 6 vs C = 14), Autonomic nerve dysfunction (I = 7 vs C = 16)
Wang GL 2009 [27]	66/66	I:24/40; C:28/38	I:63.5 ± 2.3; C:64.5 ± 3.4	Chai Hu Shu Gan San + Paroxetine	Paroxetine	Y	6 weeks	N	7.2(2.1) vs 10.1(1.7)	62/66 vs 52/66	NR
Ji XL 2013 [28]	30/30	I:18/12 C:17/13	NA	Chai Hu Shu Gan San	Fluoxetine	Y	30 days	N	9.1(3.2) vs 13.3(3.5)	25/30 vs 19/30	NR
Zhang FH 2013 [29]	40/40	I:18/22 C:16/24	I:66.3; C:65.8	Chai Hu Shu Gan San + Deanxit	Deanxit	Y	6 weeks	N	14.2(2.1) vs 17.3(2.6)	36/40 vs 31/40	NR
Ren MJ 2015 [30]	36/36	41/31	58.6 ± 2.1	Chai Hu Shu Gan San	Fluoxetine	Y	30 days	N	8.2(2.6) vs 14.1(2.8)	35/36 vs 30/36	Dry mouth, nausea, anorexia, fatigue (I = 0 vs C = 4)
Postnatal Depression											
Zhao XP 2006 [31]	45/42	I:0/45; C:0/42	I:29.04 ± 3.99; C:29.12 ± 4.26	Chai Hu Shu Gan San + Fluoxetine	Fluoxetine	Y	4 weeks	N	9.18(5.72) vs 11.36(5.73)	43/45 vs 39/42	Nausea, appetite descent, anxiety, somnipathy, quiver (Case unknown).
Zhao Y 2016 [32]	41/42	I:0/41; C:0/42	I:28.94 ± 5.03; C:30.12 ± 4.3	Chai Hu Shu Gan San + Fluoxetine	Fluoxetine	N	8 weeks	N	8.31(2.05) vs 13.96(2.16)	NR	Dry mouth, dizziness, nausea, tiredness, somnolence, quiver (I = 3 vs C = 10)
Cancer and Depression											
Fang XH 2013 [33]	45/45	I:17/28; C:24/21	I:42.3 ± 18.1; C:47.6 ± 16.9	Chai Hu Shu Gan San	Fluoxetine	N	6 weeks	N	11.78(3.21) vs 13.98(2.12)	38/45 vs 34/45	Dry mouth (I = 2 vs C = 3), constipation (I = 2 vs C = 0), dizziness and headache (I = 3 vs C = 2),insomnia (I = 4 vs C = 1), gastrointestinal dysfunction (I = 7 vs C = 2), blurred vision (I = 2 vs C = 0)
Epilepsy and Depression											
Huang XB 2015 [34]	62/60	64/58	37.28 ± 7.29	Chai Hu Shu Gan San	Paroxetine	N	12 weeks	N	17.68(1.95) vs 22.12(1.9)	41/57 vs 25/54	NR
Chronic Pelvic Inflammation and Depression											
Li L 2006 [35]	38/36/38	I:0/38; C1:0/36; C2:0/38	N/A	Chai Hu Shu Gan San	C1: Paroxetine; C2: No Intervention	N	6 weeks	N	17.71(3.91) vs C1:18.55(4.51); C2: 22.00(3.91)	34/38 vs C1:28/36; C2:18/38	NR
Post-PCI and Depression											
Wang YT 2016 [36]	30/30	NA	NA	Chai Hu Shu Gan San	Deanxit	N	4 weeks	N	15.73(6.05) vs 14.77(6.84)	25/30 vs 23/30	NR
COPD and Depression											
Yang G 2011 [37]	40/40	I:24/16; C:22/18	I:62.37 ± 6.78; C:63.6 ± 7.25	Chai Hu Shu Gan San	No Intervention	N	4 weeks	N	14.59(1.12) vs 20.15(1.08)	34/40 vs 20/40	NR
Parkinson and Depression											
Ma YZ 2011 [38]	36/32	NA	NA	Chai Hu Shu Gan San + Sertraline Hydrochloride	Sertraline Hydrochloride	Y	4 weeks	N	9.2(3.6) vs 12.3(5.4)	NR	NR
Zhou R 2016 [39]	36/36	I:18/18; C:16/20	NA	Chai Hu Shu Gan San + Duloxetine	Duloxetine	Y	4 weeks	N	15.96(3.96) vs 20.28(3.56)	NR	Nausea (I = 2 vs C = 2), headache (I = 0 vs C = 1)
Yang MJ 2010 [40]	30/30	I:18/12; C:17/13	I:62 ± 6.53; C:62.13 ± 5.92	Chai Hu Shu Gan San + Paroxetine	Paroxetine	Y	8 weeks	N	9.02(1.24) vs 13.12(2.72)	27/30 vs 21/30	Dry mouth (I = 2 vs C = 5); fatigue (I = 1 vs C = 3); Nausea (I = 2 vs C = 3); poor appetite (I = 4 vs C = 5); insomnia (I = 2 vs C = 5); constipation (I = 2 vs C = 5)
Piman syndrome and Depression											
Qiu ZJ 2012 [41]	36/36/36	I:17/19; C1:16/20; C2:17/19	I:36.17 ± 13.29; C1:36.17 ± 11.29; C2:38.83 ± 11.94	Chai Hu Shu Gan San	C1:Deanxit; C2: No Intervention	N	6 weeks	N	6.42(3.68) vs C1:5.42(4.14); C2:16.03(4.34)	NR	Mouth odor, nausea, vomit, inappetence, gastrointestinal dysfunction, diarrhea, constipation in three groups, (Case unknown)
Cerebrovascular disease and Depression											
Yao K 2013 [42]	38/38	I:20/18; C:19/19	I:65.27 ± 8.35; C:66.31 ± 7.94	Chai Hu Shu Gan San + Mirtazapine	Mirtazapine	Y	4 weeks	N	12.53(3.17) vs 15.87(3.62)	34/38 vs 30/38	Dry mouth, nausea, constipation (Case unknow)
Shang GM 2014 [43]	29/29	I:17/12; C:16/13	I:63.38 ± 10.21; C:62.91 ± 9.83	Chai Hu Shu Gan San	Fluoxetine	N	4 weeks	N	NR	27/29 vs 23/29	NR
Gastroesophageal Reflux Disease and Depression											
Zheng YJ 2016 [44]	43/42	I:18/25; C:17/25	I:32.3 ± 12.6; C:44.2 ± 7.4	Chai Hu Shu Gan San	No Intervention	N	8 weeks	N	9.2(1.3) vs 14.3(1.8)	40/43 vs 32/42	Nausea (I = 1 vs C = 5), dry mouth (I = 0 vs C = 2), dizziness (I = 0 vs C = 4)
Coronary heart disease and Depression											
Liu YH 2014 [45]	25/24	I:14/11; C:13/11	I:60.7 ± 13.6; C:56.8 ± 10.2	Chai Hu Shu Gan San	No Intervention	N	4 weeks	N	NR	19/25 vs 9/24	No adverse effect reported in both groups
Rheumatoid arthritis and Depression											
Chen PY 2015 [46]	34/34	NA	NA	Chai Hu Shu Gan San	Paroxetine	N	6 weeks	N	10.68(6.83) vs 19.31(7.69)	NR	NR
Diabetes and Depression											
Yang YL 2013 [47]	40/38	I:18/22; C:15/23	I:38.70 ± 11.10; C:37.5 ± 11.2	Chai Hu Shu Gan San	Paroxetine	Y	3 months	N	14.12(7.84) vs 22.69(11.66)	38/40 vs 28/38	NR
Cardiac neurosis and Depression											
Pei GX 2013 [48]	60/60	I:18/42; C:24/36	I:42.58 ± 6.12; C:44.32 ± 4.58	Chai Hu Shu Gan San + Deanxit	Deanxit	Y	8 weeks	N	11.42(3.45) vs 13.68(2.74)	57/60 vs 53/60	NR

**Note: **Y---Yes; N---No; I---Interventional Group; C---Controlled Group; F---Female; M---Male; HAMD---Hamilton Depression Scale; SD---standard deviation; NR--- not reported

### Methodological quality

**Fig. 2:**
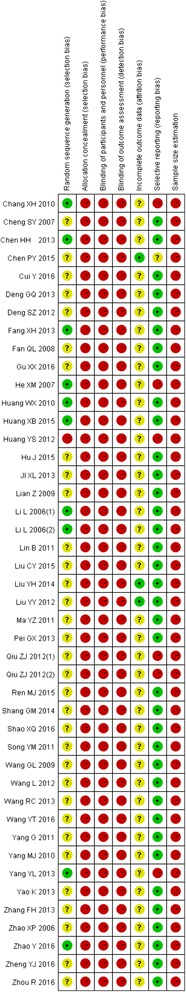
Risk of bias summary

In thisreview, we employed a quality standard of RCT evidence recommended by the Cochrane Collaboration to assess the risk of bias in the included studies. The studies included in this review were all assessed, and determined to contain high risks of bias, and were subsequently considered to be of low quality. None of the included articles contained details regarding placebos, blinding methods, or concealment of random sequences. Nine studies used a random number table for random allocation [7, 8, 11–14, 16, 28, 38]. And one article used random allocation based on whether the patient’s bed number was odd or even was considered to have a high risk of bias [15]. The other studies only mentioned “random allocation”, and their risks of bias were considered to be “unclear”. Only two study reported a withdrawal from the trial [19, 31], and the bias of incomplete outcome reporting in the article was considered to have a “low risk”, while the other studies did not provide clear information, and their biases of “incomplete outcome” reporting were considered to be “unclear”. None of the included studies registered their protocols. By comparing the predicted outcome indices in the studies—estimated based on the methodology section in the articles—with the real reported outcome indices in the same studies, this review assessed whether there existed any risk of selective outcome reporting. It was found that five studies showed inconsistencies between the predicted outcomes and the reported outcomes, and therefore, were considered to have a “high risk” of bias, while the other studies were considered to have a “low risk” of bias. The methodological qualities of the included studies are shown in Fig. 2.

### Efficacy of Chai Hu Shu Gan San

#### HAMD evaluation

**Fig. 3:**
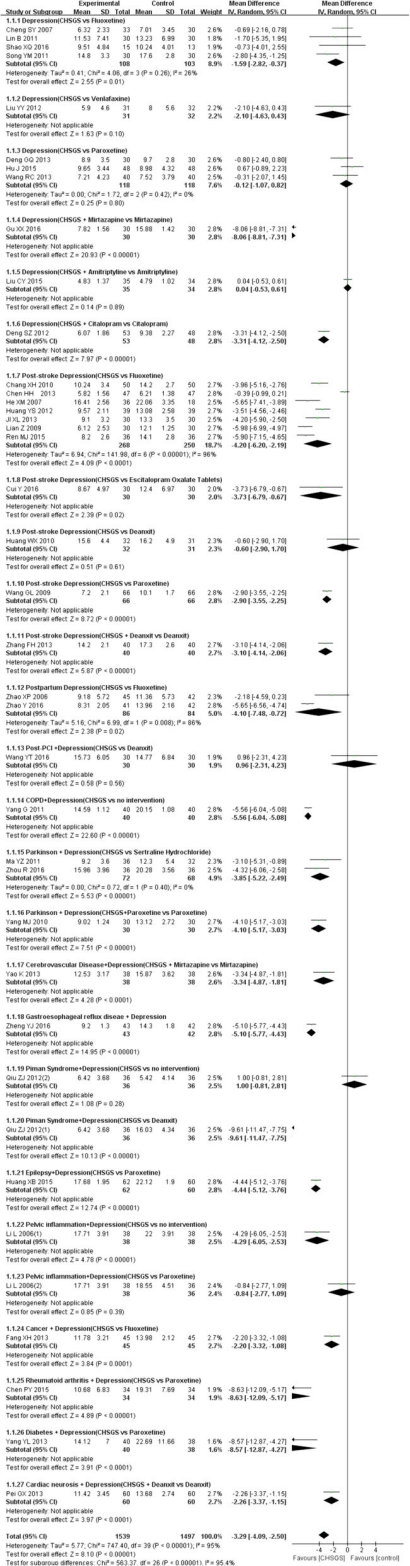
Forest plot for HAMD improvement after treatment of depression with Chai Hu Shu Gan San

Thirty-eight of the reviewed studies reported HAMD data. Total meta analyses showed better effect of Chai Hu Shu Gan San than controls (MD = − 3.29, from − 4.09 to − 2.50, I^2^ = 95%), and subgroup meta analyses also showed it favorites compared to fluoxetine for pure depression (MD = − 1.59, from − 2.82 to − 0.37, 4 trials, I^2^ = 26%), and better than fluoxetine for post-stroke depression (MD = − 4.20, from − 6.20 to − 2.19, 7 trials, I^2^ = 96%), and better than fluoxetine for postpartum depression (MD = − 4.10, from − 7.48 to − 0.72 7 trials, I^2^ = 86%). More details regarding the HAMD score improvement in depression treatment with Chai Hu Shu Gan San are given in Fig. 3.

#### Efficacy rate

**Fig. 4:**
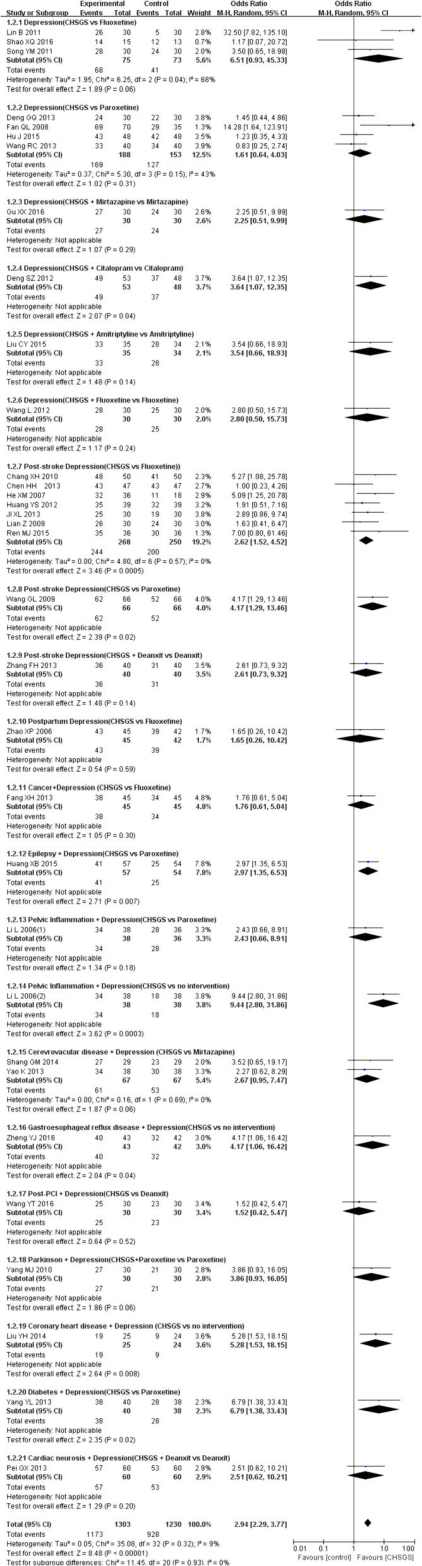
Forest plot for efficacy rate of Chai Hu Shu Gan San in treating depression

There were 33 studies reporting the efficacy rate of Chai Hu Shu Gan San for treating depression. Total meta-analysis showed that the Chai Hu Shu Gan San had a significantly higher efficacy rate than controls (OR = 2.94, from 2.29 to 3.77, I^2^ = 9%), and subgroup meta analyses showed it better effect than fluoxetine alone for treating pure depression (OR = 6.51, from 0.93 to 45.33, 3 trials, I^2^ = 68%), and also for treating post stroke depression (OR = 2.62, from 1.52 to 4.52, 7 trials, I^2^ = 0%,). Figure 4 shows the efficacy rate of Chai Hu Shu Gan San for treating depression.

#### Adverse events

None of the included studies reported severe adverse events of Chai Hu Shu Gan San. Among the 21 studies that compared the therapeutic effects between Chai Hu Shu Gan San alone and regular Western medicines for treating depression, thirteen of them did not report the safety indices. The other studies reported drug-induced symptoms such as nausea, dry mouth, and dizziness, and the symptoms in the test group treated with Chai Hu Shu Gan San were fewer than those in the control group (dry mouth: OR = 0.17, from 0.05 to 0.53, 4 trials, I^2^ = 13%; nausea: RR = 0.04, from 0.02 to 0.37, two trials, I^2^ = 0%, FEM). Among the 21 studies that compared the therapeutic efficacy of a combination of Chai Hu Shu Gan San and a Western medicine with that of the Western medicine alone for treating depression, seven did not report safety indices, reported that “there was no adverse effect in the two groups”, and the others reported many adverse effects, however, with inconsistencies in the assessment methods. Therefore, it was impossible for us to conduct a further quantitative analysis of their data. However, all of the aforementioned studies that reported adverse effects noted that the side effects in a Chinese-Western medicine combination group were fewer than those in a Western medicine group, and the side effects in both groups were likely to be induced by the same Western medicine used in each group.

## Discussion

Depression is not a simple disease. It is a neural disorder involving many pathological factors and pathophysiological symptoms. Patients with depression included in this review suffered from12different types of diseases. The meta-analysis indicated that a combination of Chai Hu Shu Gan San and fluoxetine was better than fluoxetine alone for treating PSD and PD. Meanwhile, no exact data indicated Chai Hu Shu Gan San has fewer adverse effects than regular Western medicines, so the safety of Chan Hu Shu Gan San in treating depression is worthy to be further investigated.

Chai Hu Shu Gan San is composed of 3 key herbs: Chai Hu (*Bupleurum Chinese*), Xiangfu (*Cyperus rotundus*),and Chuanxiong (*Ligusticum chuanxiong*). *Bupleurum Chinese*iswell known for its anti-inflammatory actions [49] and neuroprotective effects [50]. It has also been explored extensively for antioxidant, anticancer, and apoptotic properties in treating other diseases [51, 52]. Recently, *Cyperus rotundus*has been found to have anti-inflammatory activity in both the peripheral and central nervous system [53–55]. Moreover, *Ligusticum chuanxiong* is a classic herb with anti-inflammatory effects in treatingcardiovascular and cerebrovascular diseases [56, 57], and its conventional activity tends to be neuroprotectivein a translational medicine perspective [58, 59]. Depression has serious neurological symptoms, and theoretically, Chai Hu Shu Gan San, because of the above 3 key herbs, is a better choice for the treatment of the disease.

There are some limitations. First, the methodological qualityof the included studies was not high. Therefore, the studies were likely to have certain degrees of subjective bias. Second, the included studies in this review did not have long-term follow-up data. Therefore, we were unable to assess the relapse rate and long-term quality of life of the patient. Third, patients with depression in the included studies had many different kinds of diseases, involving many diseases and conditions associated with depression, and they were subjected to different interventions and control measures. Therefore, many types of meta-analyses would be subjected to a large degree of limitations. For these reasons, our review could not pool all the data together to conduct meta-analyses. Fourth, there existed high heterogeneity in the conducted meta-analyses, likely due to differences in the approaches that were employed by the different studies for the assessment of depression degree, primary diseases, and HAMD scores. Fifth, all of the included studies employed different assessment standards for assessing efficacy rates, and the investigations that took into consideration the improvement in primary diseases when defining efficacy rates were not included for analysis in this review. Our review only focused on the efficacy rates that were defined primarily based on the improvement in the depression symptoms. Sixth, the reporting of safety indices in the articles was not standard. Because the recruited patients in the different studies had different conditions, and because the patients were treated with different regular Western medicines in addition to Chai Hu Shu Gan San, a variety of adverse effects were observed. Moreover, the adverse effects were presented in different ways. Therefore, it was not possible to provide a quantitative assessment of all safety indices for Chai Hu Shu Gan San. Finally, all the trials were conducted in China mainland and published in Chinese, this may involve publication bias.

Here, we have to discuss the research designs of intervention and comparison. A new intervention is usually pre-estimated effective and safe. However, investigators cannot draw a definite conclusion without a correct comparison design. To avoid measurement bias, Chinese medicine clinical researchers have to focus on research design, especially with respect to intervention and comparison, for further analysis. Most trials included in this review adopted the Chinese medicine or integrative medicine intervention group compared with the western medicine controlled group, which increased the possibility of measurement biases and resultant incorrect conclusions. Hence, in further research, clinical investigators should focus on the intervention and comparison design and try to adopt Chinese placebos in the controlled group. This can reduce the possibility of measurement bias and provide reasonable clinical evidence.

It has been suggested that in the future, a meticulously designed, large-scale, multi-center RCT should be conducted to further verify the therapeutic efficacy of this Chinese medicine before a more reliable conclusion may be finally obtained.

## Conclusions

This study found that Chai Hu Shu Gan San may have some advantages in treating depression, especially PSD and PD. A meticulously designed and conducted RCT is urgently needed to further determine its safetyand efficacy.

## Abbreviations

CI: Confidence interval
CNKI: China National Knowledge Internet
COPD: Chronic obstructive pulmonary disease
FEM: Fixed-effects model
HAMD: Hamilton depression scale
MD: Mean difference
MDD: Major depressive disorder
PCI: Post-percutaneous coronary intervention
PD: Post-partum depression
PSD: Post-stroke depression
RCT: Randomized controlled trial
REM: Random-effects model
RR: Relative risk
